# Data-centric automated approach to predict autism spectrum disorder based on selective features and explainable artificial intelligence

**DOI:** 10.3389/fncom.2024.1489463

**Published:** 2024-10-21

**Authors:** Asma Aldrees, Stephen Ojo, James Wanliss, Muhammad Umer, Muhammad Attique Khan, Bayan Alabdullah, Shtwai Alsubai, Nisreen Innab

**Affiliations:** ^1^Department of Informatics and Computer Systems, College of Computer Science, King Khalid University, Abha, Saudi Arabia; ^2^College of Engineering, Anderson University, Anderson, SC, United States; ^3^Department of Computer Science and Information Technology, The Islamia University of Bahawalpur, Bahawalpur, Pakistan; ^4^Department of AI, College of Computer Engineering and Science, Prince Mohammad Bin Fahd University, Al Khobar, Saudi Arabia; ^5^Department of Information Systems, College of Computer and Information Sciences, Princess Nourah bint Abdulrahman University, Riyadh, Saudi Arabia; ^6^Department of Computer Science, College of Computer Engineering and Sciences, Prince Sattam bin Abdulaziz University, Al-Kharj, Saudi Arabia; ^7^Department of Computer Science and Information Systems, College of Applied Sciences, AlMaarefa University, Riyadh, Saudi Arabia

**Keywords:** autism spectrum disorder, data-centric analysis, autism educational planning, feature engineering, chi-square features, multi-model learning

## Abstract

Autism spectrum disorder (ASD) is a neurodevelopmental condition marked by notable challenges in cognitive function, understanding language, recognizing objects, interacting with others, and communicating effectively. Its origins are mainly genetic, and identifying it early and intervening promptly can reduce the necessity for extensive medical treatments and lengthy diagnostic procedures for those impacted by ASD. This research is designed with two types of experimentation for ASD analysis. In the first set of experiments, authors utilized three feature engineering techniques (Chi-square, backward feature elimination, and PCA) with multiple machine learning models for autism presence prediction in toddlers. The proposed XGBoost 2.0 obtained 99% accuracy, F1 score, and recall with 98% precision with chi-square significant features. In the second scenario, main focus shifts to identifying tailored educational methods for children with ASD through the assessment of their behavioral, verbal, and physical responses. Again, the proposed approach performs well with 99% accuracy, F1 score, recall, and precision. In this research, cross-validation technique is also implemented to check the stability of the proposed model along with the comparison of previously published research works to show the significance of the proposed model. This study aims to develop personalized educational strategies for individuals with ASD using machine learning techniques to meet their specific needs better.

## 1 Introduction

Autism spectrum disorder (ASD), a type of neurodevelopmental condition, has garnered considerable attention over the past few decades. It is one of the neuropsychiatric disorders that begin in childhood, and impacts development, communication, and social relationships throughout life (Ferreri, 2014). The following ways describe the complexity of ASD based on genetic predisposition, pathophysiological mechanisms, and influence of the environment (Mughal et al., 2022). ASD is characterized by a lack of quantitative abnormalities in social functioning and interaction, but an absence of development of, for instance, the ability to comprehend nonverbal communication systems such as faces and gestures; impaired capacity to establish friendships; absent spontaneous peer approach; and, nonautomatic mutuality, respectively. Further, ASD is manifested in qualitative alterations of communication, such as language developmental delays, inability to initiate and maintain conversation with other people, and repetitive and stereotyped speech patterns.

As the above discussions show, the earlier the ASD is identified, the more opportunities for positive changes in the lifestyle can be offered, although there is no cure for this condition at the moment. Early identification of ASD in children may hold some advantages since the plasticity of a child's brain may help in enhancing his or her abilities to interact with other people. It has been established that early medical assessment of these children before the age of two years gives them higher IQ as compared to those who receive this diagnosis later (Zuckerman et al., 2021). Unfortunately, recent research (Goh et al., 2016) highlights that the majority of children with ASD do not receive a diagnosis until they are at least three years old (Speaks, 2011; Asghar et al., 2022).

Exploring brain imaging as an alternative to traditional behavioral methods can offer valuable insights. However, reliance on structural images may not be entirely dependable, given the considerable variability in the developmental pace of children during their early years. As highlighted by Hussain (2021), each child's developmental trajectory is inherently unique, leading to variations in reaching developmental milestones at different ages. However, the resting-state networks that are functional begin to appear prior to birth and can be detected as early as 26 weeks of prenatal fetal age (Haartsen et al., 2016). These networks represent neural interactions that have been verified to be successful in autism detection with a precision of 60%, to about range 70% in heterogenous environments using ML algorithms (Benabdallah et al., 2018, 2020; Abraham et al., 2016). The utilization of DL methodologies has exhibited an additional enhancement in detection accuracy, achieving 80% (Epalle et al., 2021; Kashef, 2022).

Throughout the years, various tools have been recommended by researchers to diagnose ASD. These diagnostic instruments should be designed in a manner that provides health professionals with more insight and assists in arriving at correct conclusions during the diagnostic process. With the help of adaptive scales applied together with subjects' historical information a complete diagnosis is made (Kim and Lord, 2012). Of all the statistically significant activities targeted at 24–35-month-old kids, Stat includes interactive objects in 12 of them to assess social-communicative behavior. This tool forges observation on concrete behaviors that can be rooted out to have progressive predictive indices mainly on the autism scale among children aged 24–35 months. This tool is a direct observation of important and potentially occurred behaviors; therefore, it has some proof of high predictive indicators, especially for the aim to distinguish children with Autism Spectrum Disorders from those without ASD between the ages of 24–35 months (Stone et al., 2004). On the other hand, the ADOS consists of specific procedures on how to observe and assess, social—communication behaviors in people. This tool involves structured processes for interacting with specific targeted behaviors, providing a quality rating of behaviors. NICE strongly recommends the Autism Diagnostic Observation Schedule (ADOS) as an objective and effective assessment tool, offering sufficient predictive capability for diagnosing ASD (Adamou et al., 2018).

As it was seen, even after the recent developments in the diagnosis of ASD, some problems remain unresolved. Such difficulties are proved by reports that described problems that arise from the instruments: their methodologies differ across regions or countries due to cultural diversity affecting the social norms and communication (Sritharan and Koola, 2019). Thus, the goal of this study is to address these challenges with the help of a two-phase strategy. The first phase aims to precisely identify ASD through subject analysis, while the second phase aims to determine suitable teaching techniques for children with ASD. The primary contributions of this study are,

- To promote diversity within the ASD data for toddlers dataset, a new dataset called Diverse ASD Screening Data for Toddlers is generated by combining two datasets obtained from different geographical areas.
- A novel XGBoost 2.0 is utilized in this paper for predicting ASD in the toddlers with Chi-square (CHI2) features.
- Two other feature selection techniques are investigated including bidirectional elimination (BEFS), and principal component analysis (PCA).
- The evaluation of children with ASD in terms of their verbal, behavioral, and physical abilities should guide the creation of a successful teaching strategy is also analyzed in this paper.
- In this research, cross-validation technique is also implemented to check the stability of the proposed model along with the comparison of previously published research works to show the significance of the proposed model.

The study's remaining sections are arranged as follows: Section 2 provides a general summary of current literature related to employing machine learning (ML) models for ASD prediction. Section 3 outlines the dataset, delineates the study's methodology, proposes details on the ML classifiers used in the investigation, and talks about the parameters for evaluation. Moving on to Section 4, the experimental results are presented, followed by the applicability of the proposed methodology and its alignment with research objectives. Section 5 concludes the study by emphasizing conclusions and their implications.

## 2 Literature review

ASD demonstrates that human brain neural variations lead to developmental disorders. Experts in the field claim that a number of simultaneous elements operate to cause ASD. Diagnosing ASD proves to be challenging. Physicians rely on psychological and observational methods for assessment, where they appraise different areas of daily activities that may point to signs of ASD. Raj and Masood (2020) explore the potential application of ML and DL models in predicting and analyzing ASD in a variety of age groupings of people, including kids, teens, and adults. The assessment of the proposed techniques involved the use of three publicly accessible non-clinical ASD datasets. NB, LR, KNN, SVM, and ANN were used in the analysis and treatment of missing value which included imputation. According to the results of the conducted research, the CNN-based prediction model has a higher accuracy than other types of models in all three data sets. Thus, promoting and widely applying the ASD data was represented by the works of Erkan and Thanh (2019) to allow diagnosing early, using a quick, easily applicable yet comfortable instrument. In children, adults, and adolescents for ASD three different datasets are employed. The authors used SVM, KNN, and RF to the classification of the ASD data. Considering the experiments, 100 random samples were selected for classification techniques' assessment. A review of the outcomes that have been obtained from this study showed that it is possible to classify ASD using SVM and RF as viable methodologies. Unlike RF, GNB showed a maximum accuracy of 96% thus ranking it slightly lower than RF in terms of accuracy in categorizing the sets of data.

Farooq et al. (2023) proposed a method specifically designed for autism identification, employing localized training of two classifiers for ML, LR, and SVM. These classifiers are responsible for categorizing ASD factors in both children and adults. The best way to identify ASD in various age groups is determined by training a meta-classifier on these data, which are transmitted to a central server. Utilizing four distinct ASD patient datasets, each comprising over 600 records of affected children and adults, features were extracted. The proposed SVM model exhibited a 98% precision in predicting ASD in children and an 81% accuracy in adults. However, in the prediction of ASD at an early stage, Amrutha and Sumana (2021) in this context relies on ML. The study incorporated ML models, including NB, KNN, DT, and LR. The study outcome shows that the DT model obtained a 100% accuracy score. However, the process of DT takes a relatively longer time compared to other algorithms.

In recent years, the application of optimization algorithms and hybrid approaches has gained prominence in addressing complex real-world problems (Arun and Muthuraj, 2024). Similarly, Basu and Mandal (2024) presents a hybrid firefly algorithm to enhance XGBoost tuning, The role of feature selection in machine learning is explored in Raj and Singh (2024), where a genetic algorithm-based hierarchical approach is also producing good results. Furthermore, XAI techniques are being applied (Dobrojevic et al., 2023) to give better understanding with enhanced sine cosine metaheuristics and hybrid machine learning models. More popular classification technique presented by Singh and Kumar (2024) gives a solution by utilizing metaheuristic-tuned extreme learning machines (ELM).

The author in Ravindranath and Ra (2018) proposed a methodology aimed at enhancing the accuracy of ASD identification by employing minimal feature subsets. A child dataset with 292 instances and 21 features was taken from the UCI ML repository and used in the investigation. The evaluation was conducted through a binary firefly feature selection wrapper utilizing swarm intelligence. To discern between ASD and non-ASD class types, the author used this feature selection wrapper to identify 10 features out of the 21 in the ASD dataset. Dimensionality reduction was then used, and different ML models NB, DT, KNN, SVM, and MLP were used to classify ASD and non-ASD class types after feature selection. By selecting optimal features for classification model training with minimal behavioral sets, the approach demonstrated average accuracy ranging from 92.12 to 97.95%, thereby demonstrating the performance of the classifiers.

Mohanty et al. (2021) presented a method of ASD detection using a deep classifier in a stepwise manner. Initial feature identification demystifies features related to ASD to increase the efficiency of screening processes. Isolation of the ASD class type is followed by assessment criteria determination as a result of the ML models. This analysis discusses how the application of principal component analysis (PCA) may be incorporated to reduce the feature dimensions, and a deep neural network (DNN) used to identify the ASD patients. Performance results obtained based on experiments prove that the application of PCA and DNN together can provide clinically acceptable results in ASD recognition, thereby expediting the identification process.

In the context of the above study (Abdullah et al., 2019), a system with ML's aim proposes to classify people with ASD within the system. Chi-square and LASSO are used for feature selection purposes to get the important attributes in this particular study. The above-listed features make up the input data that feed the ML algorithms to help achieve the correct classification of ASD based on the above authors recognize significant traits. The findings of the study also show that the LR has achieved better accuracy as compared with other learning models and the percentage is 97.54% accuracy using CHI2 as the feature selection method; In the same regard, Alwidian et al. (2020) used the ML to predict ASD. For this purpose, the authors used various types of ML models in this study. The study also reveals that the proposed ML model WCBA in most of the scenarios, has an accuracy of 97%.

Kumar and Sree proposed a DL system to diagnose and diagnose ASD in Raj and Masood (2020). To evaluate the system's performance, the investigation utilized three datasets. The findings revealed that the CNN model attained the highest accuracy, reaching 99.53%, particularly on the children dataset. Alkahtani et al. (2023) proposed a TL paradigm based on face landmarks for children with ASD. Additionally, they compared the learning models' performances using ML models. The result of the study shows that MobileNetV2 achieved the highest accuracy of 92% for ASD prediction.

From the above-described works, it is clear that investigating the ability of DL models is needed for the identification of ASD in the human population. The above-mentioned works are mostly based on traditional ML approaches and report varying performance for ASD detection. The complete summary of state-of-the-art recent related works is shown in Table 1. The performance of these models can be further improved. To facilitate this, the performance of several ML models has been compared with that of the ensemble ML model in this work. For each of the separate population sets, individual models were prepared and compared.

**Table 1:** Summarization of related work.

**References**	**Classifiers**	**Dataset**	**Performance**
Raj and Masood (2020)	SVM, NB, LR, KNN, ANN, and CNN	UCI	99.53% for adult, 98.30% for Children, 96.88% for adolescents with CNN
Erkan and Thanh (2019)	KNN, SVM, RF	AQ-10	100% RF and SVM
Farooq et al. (2023)	SVM, LR	UCI	98% Children dataset, 81% adult dataset using SVM
Amrutha and Sumana (2021)	NB, KNN, DT, LR	Kaggle	98% DT
Ravindranath and Ra (2018)	NB, J48, SVM, KNN, MLP	UCI	97.95% SVM
Mohanty et al. (2021)	DNN	Q-Chat-10 questions (same)	89.26%
Abdullah et al. (2019)	RF, LR, KNN	Brain images and EEG dataset	97.541% LR with CHI2 features
Alwidian et al. (2020)	CMAR, CBA, FACA, MCAR, FCBA, ECBA, and WCBA	UCI	97% WCBA
Raj and Masood (2020)	CNN, ANN, KNN, LR, SVM, NB	UCI	98.53% CNN
Alkahtani et al. (2023)	MobileNet, VGG-16, MLP, LR, LinearSVC, RF, DT, GBC, ADA, KNN	Kaggle's autistic children dataset	92% MobileNet

## 3 Materials and methods

This section covers a comprehensive examination of the entire study, comprising an analysis of the datasets used to identify autism and the analysis of the best ways to teach after studying the conduct of children with autism. It outlines the methodology employed in the study and its implementation, offering a brief overview of the ML classifiers utilized in the research.

### 3.1 Overview of study

**Figure 1:**
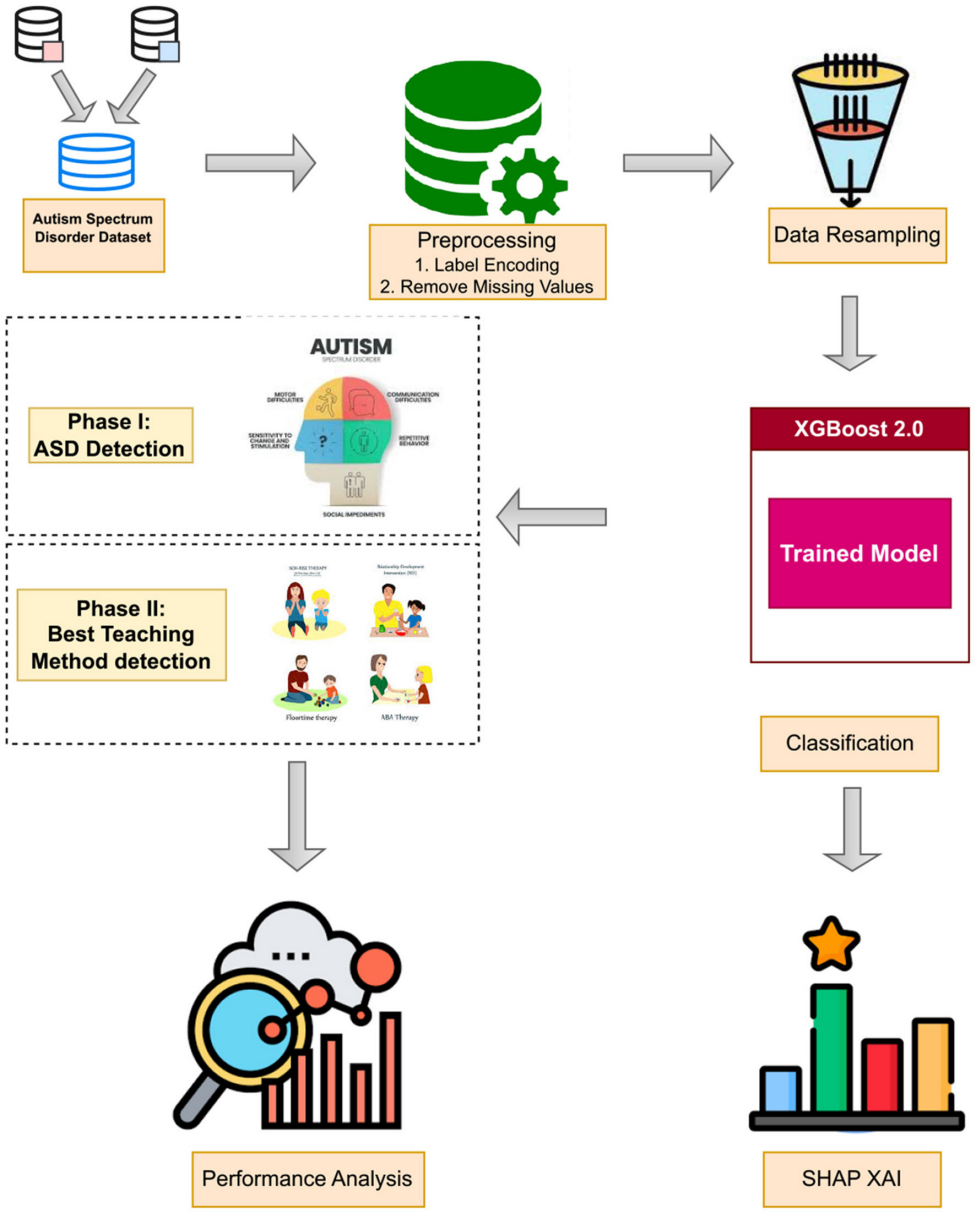
Proposed methodology for detection autism spectrum disorder in children.

The study is divided into two phases. The initial phase primarily centers on the diagnosis of ASD, employing both statistical and ML methods on a dataset specific to ASD. The first step refers to the initial preprocessing in which categorical data has been changed into numerical format, whereas the SMOTE technique is used for imbalanced-dataset problems. Hence, three feature selection strategies are employed to pinpoint the most impactful characteristics, aiming for ML models performance enhancement. The ML models utilized for ASD detection include extreme gradient boosting (XG), gradient boosting machine (GBM), KNN, RF, DT, LR, SVM, and XGBoost 2.0. Furthermore, an analysis of feature ranking is carried out to demonstrate the importance of these detected features. This is graphically represented in Figure 1. The hyper-parameteres of all learning models are shared in Table 2. The complete pseudocode is shared in Algorithm 1.

**Table 2:** Hyper-parameteres values of all learning models.

**Model**	**Hyper-parameters**
SVM	Kernel =“RBF”, Gamma =“0.01”, C =“1.0”, Degree = 3
RF	trees = 100, maximum_depth = 15, minimum_sample_split = 2
DT	criterion =“gini”, maximum_depth = 15, minimum_sample_split = 2
GBM	learning rate =“0.1”, trees = 100, maximum_depth = 15
LR	penalty =“l2”, C =“1.0”, solver =“liblinear”, maximum_depth_iteration = 100
XG	learning rate =“0.05”, trees =“150”, maximum_depth = 6
KNN	no. of neighbors = 5, weights = distance, metric = minkowski, p = 2
XGBoost 2.0	learning rate =“0.02”, trees = 250, maximum_depth = 8

**Algorithm 1:**
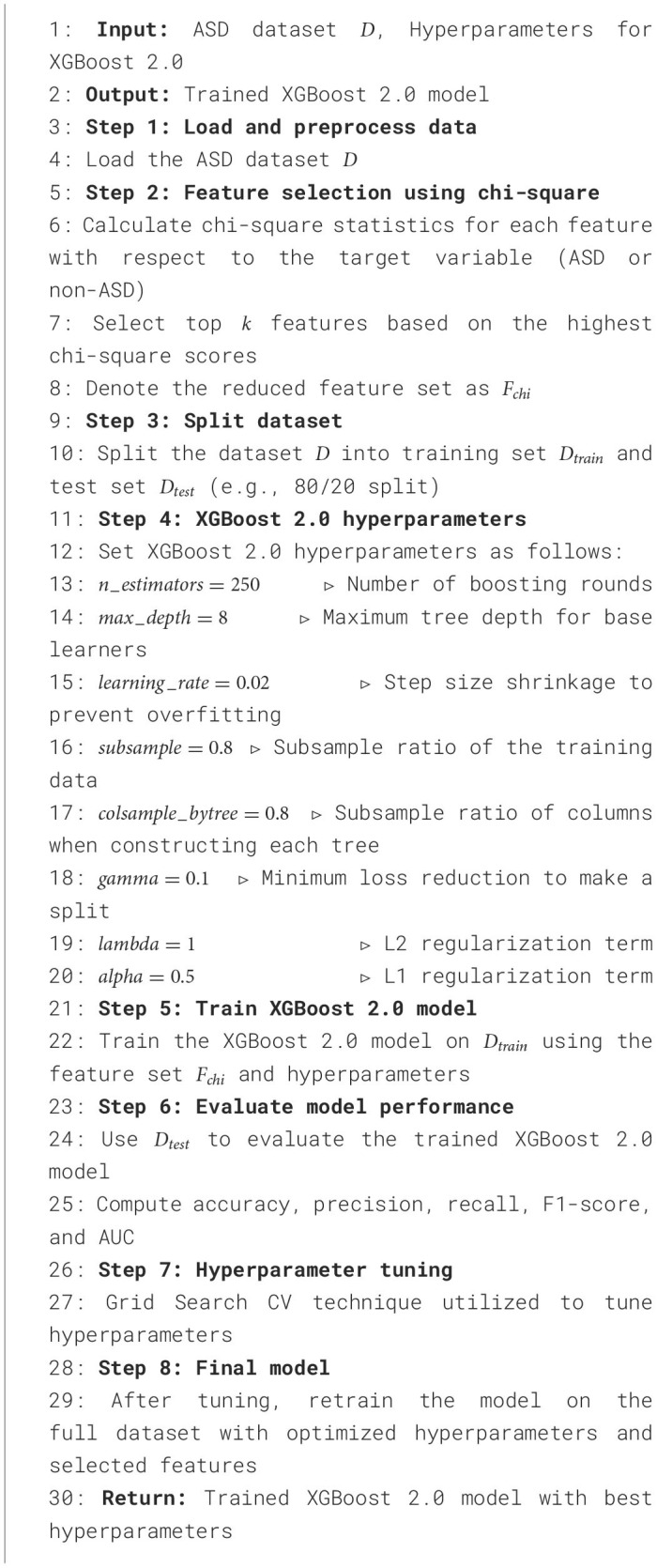
XGBoost 2.0 with chi-square feature selection for ASD dataset.

**Figure 2:**
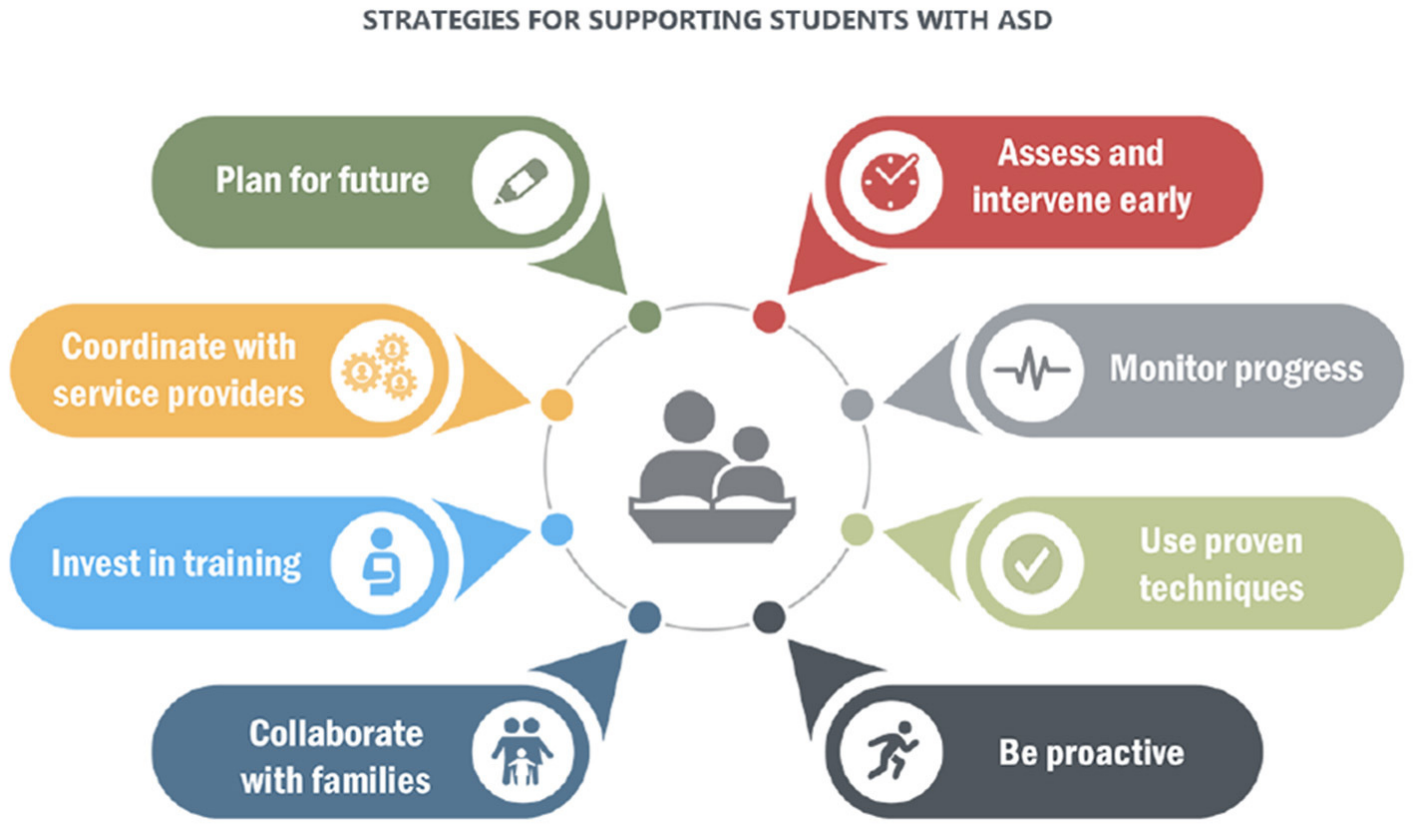
Teaching method strategies for autism spectrum disorder children.

Phase II aims at identifying the optimal ways that should be used for educating children with ASD. For the model to arrive at this objective, it must come up with a mechanism that accepts data as an input, performs some processing on the data, or conducts ML algorithms and gives out a best fit. The teaching strategies recommended in this study are accessible below via Figure 2.

### 3.2 Datasets used for ASD

ASD is a neurodevelopmental disorder that leads to high health care costs and early detection can lead to a great savings opportunity. ASD diagnoses take too long in the times that are consumed waiting for the same and incompetence on diagnosis methods (Dataset, 2022). The rise in the incidence of ASD cases worldwide also serves as an indicator of the immediate need for creating efficient, easily applicable screening tools. As a result, there is an imminent requirement for a convenient and easy-to-reach ASD screening tool that would allow healthcare professionals to proceed with the necessary measures and give individuals useful recommendations regarding further seeking of formal clinical evaluation. With the increasing global burden of ASD, a limitation associated with the lack of datasets with behavioral phenotypes is a significant barrier to comprehensive analyzes that would improve the effectiveness, sensitivity, specificity, and predictive value of screening for ASD.

**Figure 3:**
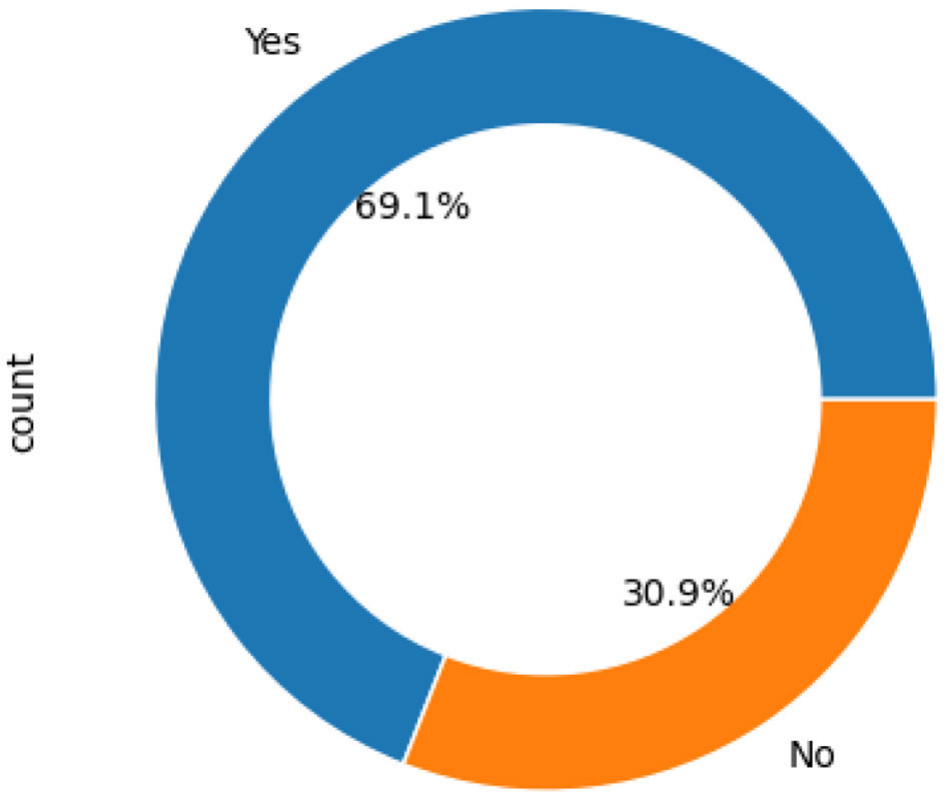
Dataset target class division.

This study combined two datasets named “Diverse ASD Screening Data for Toddlers,” which gathered information using a 10-item Q-Chat questionnaire. Binary transformation was applied to items with response options “Always,” “Never” “Usually,” “Rarely,” and “Sometimes,” in the Q-Chat-10 dataset. Specifically, a “1” was assigned to any question in the Q-Chat-10 for questions 1 through 9 (A1–A9) if the response was “*Rarely, ”* “*Never, ”* or “*Sometimes.”*. However, in question 10 (A10), a “1” was assigned if the criteria were met as outlined in Table 3. The dataset target class division is shown in Figure 3.

**Table 3:** Descriptions of the dataset used in the study.

**Attributes**	**Description**
A1	Child response on calling name?
A2	Does the child make eye contact?
A3	The child create any wishes for anything?
A4	Your child shares his/her interest with you?
A5	Your child do imaginative play, like pretending to talk on a toy phone or taking care of dolls?
A6	Does your child track the direction of your gaze?
A7	When you or another family member is visibly upset, does your child exhibit signs of wanting to offer comfort?
A8	Could you characterize your child's initial words?
A9	Child says goodbye by waving?
A10	Child gaze at something for a longer time?
Age	This indicates the age of toddlers, measured in months.
Score by Q-chat-10	It indicates Score by Q-chat-10
Sex	Gender of chiled male or female
Ethnicity	it Indicates the regions list
History of ASD in the family	Has any immediate family member been diagnosed with a Pervasive Developmental Disorder (PDD)?
Who is completing the test	Its denotes the who is responding for the questionaire
Class	it denotes the ASD Trait

### 3.3 Data on autism spectrum disorder screening for young children

Information from toddlers' ASD screenings makes up the ASD Toddler dataset (Dataset, 2022), which has significant features that could be used for further research, particularly in detecting autistic symptoms and refining the classification of ASD cases. The dataset can be accessed through Kaggle, an online marketplace for datasets. The dataset encompasses diverse information, incorporating individual characteristics and 10 behavioral elements (Q-Chat-10) that have been shown in the field of behavioral science to be advantageous for distinguishing between individuals with ASD and control groups. The dataset contains 17 columns and 1,054 occurrences.

### 3.4 Saudi Arabian toddler ASD screening data

This dataset consists of two sets of screening information gathered from toddlers aged 12–36 months across various regions of Saudi Arabia. It distinguishes between individuals diagnosed with ASD and those without it (Dataset, 2022). The data-gathering process involves administering an online questionnaire via Google Forms. This questionnaire includes the Arabic version of each Q-CHAT-10 question, along with supplementary information about respondents such as gender, age, geographical location, country or region, and family history pertinent to the screening of ASDs. The dataset encompasses 506 instances, with a total of 17 columns.

### 3.5 Data preprocessing

Typically, non-numeric labels or categories are used with categorical data, necessitating their transformation into a format compatible with machine learning models. One often used method, label encoding, gives each category in a feature a unique integer, making it easier to integrate categorical data into machine learning processes. This paper employed label encoding to convert categorical features into numerical representations in the dataset. This preprocessing step aimed to improve the adaptability of the data to various machine learning algorithms.

### 3.6 Data resampling

Unbalanced datasets, which have an imbalanced distribution of the target classes, may lead to model overfitting. Data resampling can help balance the dataset and reduce bias. Datasets of this type can be challenging to classify as models often overfit to the major class. To resolve this dilemma, several methods of data resampling have been developed. Oversampling entails increasing the representations of the minority class such that their proportion matches that of the majority class. This multiplication increases the size of the set giving rise to more features for model training, which may lead to improved performance. In this research, SMOTE is utilized as the oversampling strategy in this investigation. The SMOTE (Umer et al., 2021) method is used as an oversampling strategy to overcome unbalanced medical data. SMOTE increases the instances of minority class through random synthetic data generation using its k nearest neighbors approach based on Euclidean distance. In other cases, new variants resemble the original observations as they are engineered from original attributes. SMOTE is not considered among the most effective approaches for managing high-dimensional data since it may lead to the introduction of excess noise. This study produces a new training data set using the SMOTE method.

### 3.7 Features selection

Feature selection involves identifying important features from data that can be effectively utilized to train ML algorithms or generate derived features. Researchers have found that feature selection can increase the ML models' effectiveness. The prevalent adage in ML is “garbage in garbage out.” In this proposition, meaningless data produces nonsense output. In contrast, informational data may lead to favorable outcomes. Therefore, the process of feature selection plays a crucial role in pinpointing significant attributes within raw data, ultimately enhancing the consistency and accuracy of learning algorithms. In this research, authors employed three distinct feature selection methods, BEFS, PCA, and CHI2.

#### 3.7.1 Bidirectional elimination feature selection

Linking the forward selection and backward BEFS is one of the selection subprocesses (Mao, 2004). The model performance increases by gradually adding features to empty feature set, while simultaneously eliminating features that do not contribute significantly (backward elimination). Until a consistent subset of crucial traits is achieved, this iterative procedure is continued. However, bidirectional elimination is a tool that facilitates the selection of informative features in big dataset feature spaces and improves interpretability and generalization.

### 3.8 Chi square

CHI2 stands out as a widely employed technique for feature selection, particularly in the context of text data (Yang et al., 2016). Feature selection serves the purpose of examining the independence between the presence of a particular term and the presence of a specific class. More formally, authors compute the following term scores for a forgiven document D and order them accordingly. The equation below is used to calculate Chi2 score


(1) $$\begin{array}{l} X^{2}\left(D,t,c\right)=\underset{e_{t}\varepsilon \left[0,1\right]}{\sum }\underset{e_{c}\varepsilon \left[0,1\right]}{\sum }\frac{{\left(Ne_{t}e_{c}-Ee_{t}e_{c}\right)}^{2}}{Ee_{t}e_{c}} \end{array}$$

where

- *E* is the predicted frequency, and *N* is the observed frequency,
- if term *t* appears in the paper, *e*_*t*_ = 1, else 0,
- If the document is in the *c* class, *e*_*c*_ equals 1, if not equals 0.

A high CHI2 score for each feature (term) demonstrates that the independent null hypothesis, *H*_0_ has to be rejected, this means that the occurrence of class and term are two dependent phenomena. In this case, authors need to choose the characteristic for an ASD classification.

### 3.9 Principal component analysis

PCA serves as a linear method for selecting optimal features from the provided dataset. Employing an unsupervised approach grounded in Eigenvectors analysis, PCA identifies the essential original features for the principal component (Lasalvia et al., 2022). The principal component is essentially a linear combination of optimally weighted observed features. The result of the PCA feature selection technique yields principal components, the count of which is either equal to or less than the features in the original dataset. While PCA feature selection proves beneficial in various scenarios, it is not the preferred choice in situations characterized by excessive multicollinearity.

In this research, PCA is used after transforming categorical data into continuous representations. Specifically, categorical features were encoded into binary or numerical format using one-hot encoding, and then PCA was used to extract principal components, capturing the relationships and variance between these transformed categorical features. This approach helps in reducing the high-dimensional feature space and mitigating multicollinearity, while preserving important patterns. After PCA feature selection, we have selected 11 significant features with PCA AQ1-AQ10 and Q10 chat score.

### 3.10 Machine learning models

Various ML classifiers are employed for the purpose of ASD classification. Initially, individual assessments are conducted for models such as LR, RF, SGD, XGBoost, SVM, KNN, DT, and ETC. The evaluation involves utilizing the best hyperparameter settings for each model, determined through fine-tuning. Following this initial assessment, the top-performing models are selected to create a hybrid model.

#### 3.10.1 Logistic regression

LR is an adaptive regression method, which constructs predictors as a series of binary covariates performed in the form of Boolean combinations (Ishaq et al., 2021). LR is received from the naming of the function used in the heart process of this method, the logistic function. The sigmoid function is also known as the sigmoid function. Any real-valued number can be matched by this sigmoid curve and mapped to a value between 0 and 1.

#### 3.10.2 Random forest

RF is applicable to both regression and classification problems. It operates as an ensemble classification method, relying on tree-based classifiers (Manzoor et al., 2021). Additionally, RF addresses overfitting concerns through a resampling bootstrap strategy. The voting procedure is employed to ascertain the optimal prediction estimate. In this regard, it identifies the key features in a data set and provides only a simple score for feature importance. Data reconstruction occurs in classification research and feature selection is also used to enhance the accuracy. The process of classification in the bagging approach uses boot-strapped samples for training many models. RT outperforms a DT in terms of providing a more homogenous ensemble forecast equation that provides the test statistic for a single function based on the feature selection method.

#### 3.10.3 Stochastic gradient classifier

SGD functions similarly to SVM and LR (Rustam et al., 2019). In the realm of multi-class classification, using a one-vs.-all strategy, SGD combines several binary classifiers to improve its classification power. Given the random selection of examples from the batch, precise hyperparameter values are crucial for obtaining accurate results with SGD. Notably, the algorithm exhibits high sensitivity to feature scaling.

#### 3.10.4 Support vector machine

SVM, a linear model applicable to both classification and regression, was employed for categorizing drug reviews into negative, positive, and neutral classes (Toledo-Pérez et al., 2019). SVM achieves classification by drawing multiple hyperplanes, and the one with a substantial margin separating the data is chosen. In this research, SVM utilized the “rbf” kernel with hyperparameters set to C = “1.0” and Gamma = “auto.”

#### 3.10.5 K nearest neighbor

K-NN, a non-parametric algorithm, selects the nearest neighbor to the point under prediction (Zhang, 2012). For example, within the x train set document, the algorithm identifies all neighbors of x, accounting for potential overlaps. These neighbors are then assigned scores, and only the K neighbors with the highest scores are considered significant.

#### 3.10.6 XGBoost

XGBoost (XG) stands out as a prominent machine learning algorithm employing gradient boosting and ensemble learning (Chen and Guestrin, 2016). Its core reliance on decision trees comes with built-in regularization to prevent overfitting, and it uses a one-versus-all strategy, SGD combines several binary classifiers to improve its classification power, and XGBoost excels in handling incomplete data, and boasts universal applicability. The flexibility, scalability, and open-source nature of XGBoost contribute to its status as one of the most favored choices for predictive modeling, especially in practical applications and data science competitions.

#### 3.10.7 Decision trees

The DT method is essential for identifying and predicting target labels in DT models. It starts by selecting the root entity and then moves to leaf nodes for label prediction (Manzoor et al., 2021). The Gini index and information gain are two primary techniques for selecting the root node in DT models, with information gain being the preferred method.

#### 3.10.8 Extra-tree classifier

ETC is an ensemble tree classifier, is constructed using randomized trees, forming a forest of DTs (Sharaff and Gupta, 2019). It adopts an ensemble learning approach where the final classification result is achieved by amalgamating de-correlated trees. Similar to RF, its operational principle is nearly identical, differing only in the construction of individual trees. In the case of ETC, random sampling is applied to select the K best features, while the Gini index is employed to identify the optimal feature for splitting data elements within the tree.

### 3.11 Proposed XGBoost 2.0 model

XGBoost 2.0 represents an advanced version of the popular eXtreme Gradient Boosting (XGBoost) algorithm, widely recognized for its efficiency, flexibility, and scalability in machine learning tasks (Zhang et al., 2022). This iteration introduces several enhancements, including improved handling of categorical features, accelerated training via better optimization techniques, and more robust support for distributed computing. XGBoost 2.0 also integrates new regularization methods to prevent overfitting, making it more effective in handling large-scale datasets with high dimensionality. Moreover, the introduction of additional custom loss functions allows for greater flexibility in model tuning. These innovations make XGBoost 2.0 particularly suited for complex predictive modeling tasks, outperforming many traditional machine learning models in both accuracy and speed.

### 3.12 Evaluation parameters

Evaluating the performance of a model is crucial to understanding how well it generalizes to new, unseen data. Several metrics are commonly utilized to evaluate the effectiveness of categorization models, here four key parameters are used: precision, F1 score, accuracy, and recall.

The most commonly used metric, accuracy, describes the relation of instances that have been predicted correctly to all instances in a dataset overall While accuracy gives a broad picture of the model's correctness, unbalanced datasets with a large number of one class over the others may not be a good fit for it:


(2) $$\begin{array}{l} Accuracy=\frac{\text{Number of correct predictions}}{\text{Total number of predictions}} \end{array}$$

(3) $$\begin{array}{l} Accuracy=\frac{TP+TN}{TP+TN+FP+FN} \end{array}$$

Recall (sensitivity) quantifies how well a model can identify all the relevant examples for a given class. It is the proportion of real all-positive observations to true positive ones. In situations where missing good examples is expensive or undesirable, recall is essential. The formula for the recall is:


(4) $$\begin{array}{l} Recall\left(Sensitivity\right)=\frac{TP}{TP+FN} \end{array}$$

Specifically, precision relates to the capability of a model that makes positive predictions assigning elements correctly how many observations with a positive label out of all predicted as positives are labeled correctly. It is particularly important when the cost of false positives is significant. The precision formula is:


(5) $$\begin{array}{l} Precision=\frac{TP}{TP+FP} \end{array}$$

The F1 measure provides a complete statistic that takes into account both false positives and false negatives by computing the harmonic mean of precision and recall. It proves particularly valuable in situations involving imbalanced datasets or scenarios where there is a disparate impact associated with the costs of false negative and positive results. The formula for the F1 score is:


(6) $$\begin{array}{l} F1Score=2\times \frac{Precision\times Recall}{Precision+Recall} \end{array}$$

While accuracy offers a broad indication of a model's performance, more detailed evaluation techniques include precision, recall, and F1 score of the performance due to the intrinsic presence of false positives and false negatives. The decision regarding which metric to prioritize is contingent upon the particular requirements and goals of the relevant machine learning assignment.

## 4 Results and discussion

Supervised ML techniques were employed to assess the performance of the model. A split ratio of 80:20 was applied to divide the data into training and testing sets, a widely adopted practice in various studies to tackle classification tasks, thereby mitigating the risk of overfitting. A variety of evaluation measures were used to assess the machine learning classifiers' performance. Every experiment was carried out using various libraries in a Python context. The calculations were performed using a Dell PowerEdge T620 with a 2 GB graphics processing unit, 16 GB DDR4 Random Access Memory, and 2x Intel Xeon 8 Cores running at 2.4 GHz (RAM).

This section discusses two scenarios. In the first scenario, an initial experiment is conducted using the Diverse ASD Screening Data for Toddlers Dataset to estimate the prevalence of ASD. To achieve this, a range of ML classifiers are utilized, such as LR, RF, SGD, XGBoost, SVM, KNN, DT, and ETC. An 80:20 ratio is used to split the dataset into subsets for testing and training, with 80% of the data for training the model and the remaining 20% for testing. The models are then trained using important features that were found using feature selection approaches. Their performance is then assessed on a test dataset consisting of 20% of the data. Additionally, a 10-fold cross-validation method is applied. During the second phase, the study endeavors to determine the most effective intervention treatment for children diagnosed with ASD using machine learning models. This phase employs comparable settings and datasets to those utilized in the initial experiment.

### 4.1 First scenario

#### 4.1.1 Results of original feature

Table 4 shows the results received from ML models using the full initial features. These models are characterized by high scores for all the evaluation metrics. It is worth noting that models based on trees such as LR, and SVM also did well registering accuracy scores high at 0.94. Interestingly enough, the linear and tree-based ensemble models perform well.

**Table 4:** Machine learning models results on original feature set.

**Models**	**Accuracy**	**Precision**	**Recall**	**F1 score**
LR	0.94	0.94	0.93	0.93
SVM	0.94	0.92	0.93	0.92
RF	0.93	0.95	0.95	0.95
DT	0.91	0.91	0.91	0.91
KNN	0.92	0.94	0.91	0.92
XG	0.93	0.94	0.94	0.94
GBM	0.92	0.93	0.91	0.92
XGBoost 2.0	0.95	0.94	0.96	0.95

On the other hand, linear methods, like DT for example, show poor performance because of the limitations of smaller feature sets. The obtained accuracy for the LR and SVM is 0.94. Nevertheless, when assessed using the original data set, XGBoost 2.0 is the best model obtaining an accuracy score of 0.95, a precision level of 0.94, a recall rate of 0.96, and the F1 measure stands at 0.95 above all other models used in this scenario.

#### 4.1.2 Results with bidirectional elimination feature selection

The performance of the bidirectional elimination technique for machine learning models is demonstrated in Table 5. Given the original features, the performance degradation can be noticed for all models.

**Table 5:** Bidirectional elimination results for ML models.

**Models**	**Accuracy**	**Precision**	**Recall**	**F1 score**
RF	0.92	0.91	0.91	0.91
SVM	0.92	0.91	0.91	0.92
DT	0.90	0.92	0.91	0.91
GBM	0.91	0.86	0.88	0.87
LR	0.92	0.93	0.92	0.92
XG	0.92	0.91	0.92	0.92
KNN	0.85	0.86	0.86	0.86
XGBoost 2.0	0.93	0.93	0.94	0.93

In terms of accuracy, LR, RF, SVM, and XG exhibited the highest performance with an accuracy score of 0.92, closely trailed by GBM at 0.91. DT and KNN also achieved commendable accuracy scores of 0.90 and 0.85 accuracy, respectively. The XGBoost 2.0 ensemble model outperformed other models with an accuracy score of 0.93, which is the highest when considering BEFS but lower than the original feature set. In terms of precision, XGBoost 2.0 outperformed the other models, scoring 0.93, which suggests that it can accurately identify affirmative cases. With a recall score of 0.94, XGBoost 2.0 performed best out of all the models, demonstrating its ability to detect all positive cases. Lastly, the F1 score highlights that XGBoost 2.0 continues to perform excellently overall at 0.93, taking into account both precision and recall. These findings are valuable as they offer clear insights into the choice of ML models for this task, with XGBoost 2.0 being deemed suitable after demonstrating strong predictive capabilities in autism detection.

#### 4.1.3 Experimental results with PCA feature selection

The models' performance using PCA feature selection is shown in Table 6 and highlights the significance of each model. LR stands out with an accuracy of 0.97, marking the highest performance when employing PCA feature selection. XGBoost 2.0 completes evaluation in a more considerable way since it has an accuracy score of 0.97 which ultimately predicted exceptional performances than any other model. Regarding precision, XGBoost 2.0 still retains a high score of 0.97, which means that it has the ability to implement proper classification of positive cases with few false positives. With a recall score of 0.98, XGBoost 2.0 beats all other models, demonstrating its ability to capture almost all positive cases. The F1 score strikes a balance between recall and precision, and also attests to XGBoost 2.0 exceptional overall performance with a score of 0.97.

**Table 6:** Machine learning models results with PCA features.

**Models**	**Accuracy**	**Precision**	**Recall**	**F1 score**
SVM	0.96	0.96	0.96	0.96
RF	0.96	0.98	0.97	0.98
GBM	0.91	0.94	0.94	0.94
DT	0.93	0.93	0.93	0.93
LR	0.97	0.96	0.97	0.97
XG	0.97	0.99	0.98	0.98
KNN	0.94	0.96	0.95	0.95
XGBoost 2.0	0.97	0.97	0.98	0.97

#### 4.1.4 Experimental results with CHI2 feature selection

**Figure 4:**
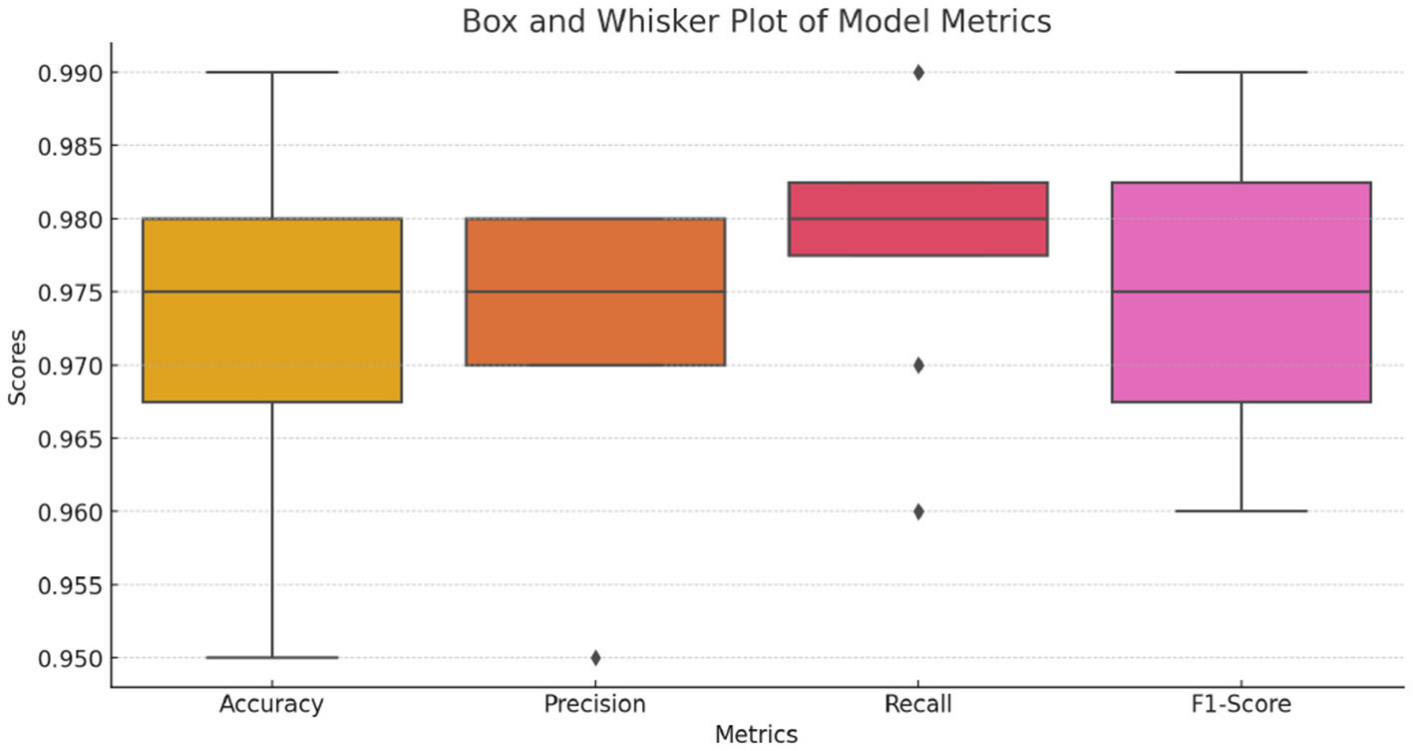
Box and whiskers' plot.

Table 7 represents models' performance with the use of the ML-based feature selection method. As per the outcomes, it can be said that there has been an enhancement in terms of SVM and LR. This is because selected features made data more linearly separable allowing SVM to establish a clear hyperplane with sufficient margin for effective classification. The box and whiskers' plot are shared in Figure 4.

**Table 7:** Machine learning models results with CHI2 features.

**Models**	**Accuracy**	**Precision**	**Recall**	**F1 score**
SVM	0.98	0.97	0.98	0.97
RF	0.98	0.98	0.98	0.98
DT	0.97	0.97	0.96	0.96
GBM	0.95	0.95	0.98	0.96
LR	0.98	0.98	0.99	0.99
XG	0.97	0.98	0.98	0.98
KNN	0.96	0.97	0.97	0.97
XGBoost 2.0	0.99	0.98	0.99	0.99

These results show that the application of the CHI2 feature selection method in this study has improved even further, compared to both BEFS and PCA methods of feature selection. In particular, XGBoost 2.0 shows the best values for all measures and demonstrates an outstanding accuracy score of 0.99 which can be interpreted as the highest prediction ability of this model. With a precision score of 0.98, it exhibits the capability to identify positive cases at the highest accuracy possible with very few false positives. In addition, the recall score of 0.99 proves that the chosen method is effective in detecting almost all positive cases, as it rarely misses them. The F1 score is a trade-off between recall and precision and also confirms XGBoost 2.0 superiority with near-perfect results of 0.99. This is to show that the ML models on CHI2 feature selection methodology have enhanced the overall capturing of positive instances as well as making accurate and precise predictions thus a greater increase in performance level. Among them, XGBoost 2.0 could be considered the best option to address the discussed task as it provides quite strong scores for various metrics. This implies that the CHI2 feature selection process found and kept the highest amount of informative features that made it possible for models to achieve outstanding results. Table 8 shows 10 folds cross-validation results. According to the cross validation findings, the proposed model has an average accuracy of 98.6%. Additionally, the F1 score, mean precision, and recall are 98.6, 98.8, and 98.7% respectively. These results consistently highlight the efficacy of the paradigm being studied.

**Table 8:** Results of proposed approach concerning cross validation.

**Fold**	**Accuracy**	**Precision**	**Recall**	**F1 score**
Fold-*1*	98.2%	98.5%	98.4%	98.5%
Fold-*2*	98.4%	98.6%	98.5%	98.6%
Fold-*3*	98.6%	98.7%	98.6%	98.7%
Fold-*4*	98.8%	98.9%	99.9%	98.8%
Fold-*5*	98.9%	98.9%	98.8%	98.8%
Fold-*6*	99.9%	98.9%	98.9%	98.8%
Fold-*7*	98.5%	98.9%	98.6%	98.7%
Fold-*8*	98.7%	98.8%	98.7%	98.8%
Fold-*9*	98.7%	98.7%	98.8%	98.8%
Fold-*10*	98.9%	98.9%	98.9%	98.9%
**Average**	**98.6%**	**98.7%**	**98.8%**	**98.6%**

### 4.2 Performance comparison

In this sub-section, the proposed model compared to appropriate findings in previous research is presented in Table 9. Previously published research works used modestly assembled ASD datasets. For example, In Abdullah et al. (2019) feature extraction methods CHI2 with a maximum accuracy of 97.541% was used. In a different study, Mohanty et al. (2021) used deep learning techniques and, achieved top marks in the accuracy category with a percentile of 89.26%. implemented the machine learning strategy using the method of characteristic selection and received the highest percentage of accuracy 98% for the children set, 81% for adult channel (Farooq et al., 2023). In a recent research Raj and Masood (2020) the ASD detection accuracy was 97%.

**Table 9:** Proposed system comparison with other studies.

**References**	**Approach**	**Performance**
Raj and Masood (2020)	CNN	96.88%
Farooq et al. (2023)	SVM	98.32% for Children dataset, 81% for adult
Ravindranath and Ra (2018)	SVM	97.95%
Mohanty et al. (2021)	DNN	89.26%
Abdullah et al. (2019)	LR	97.541%
Alwidian et al. (2020)	WCBA	97%
Shinde and Patil (2023)	MLP	94.20%
Themistocleous et al. (2024)	XGBoost	98.50%
**Proposed**	**XGBoost 2.0 with CHI2**	**99.29%**

**Figure 5:**
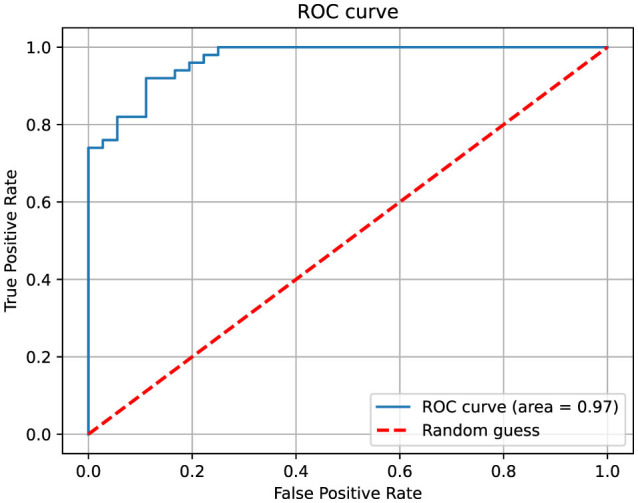
ROC-AUC curve of the proposed model.

The suggested method, applied in this study includes feature selection and is based on a combination of ensemble XGBoost 2.0 model with CHI2 feature selection. This method achieved an accuracy as high as 99.29%. This finding indicates that the developed method outperforms relatively recent studies of related works and, therefore could be considered a substantive and promising contribution to ASD classification. The ROC-AUC curve is shown in Figure 5.

### 4.3 Discussion

The ASD datasets have been the subject of numerous studies, but a lot of improvement is still needed in their predictive models. However, this study took the opportunity to collect ASD datasets and handle class-imbalance constraints by using the SMOTE technique. A number of classifiers, such as RF, LR, DT, KNN, SVM, XG, GBM, and XGBoost 2.0, were then applied in order to detect ASD in children and choose the best teaching strategy for them. After that, three feature selection methods are used i.e., BEFS, PCA factorization, and CHI2 method of feature extraction. Lastly, XGBoost 2.0 along with CHI2 provided better results than other methodologies increased the ASD detection efficiency, and detected appropriately for the optimal teaching method for children suffering from ASD.

The study results reveal several critical and relevant characteristics that can help diagnose ASD at the early stages. In particular, A8, A7, A6, and A1 were determined to be the most salient attributes from the point of view of the ML model. This in-depth inquiry highlights the adequacy of essential features for ASD identification and holds great potential for successful uses in diagnosing ASD and identifying the optimal teaching practices for children with ASD. The advancements in XGBoost 2.0 are particularly valuable for autism spectrum disorder (ASD) prediction due to the nature of ASD data, which is large, complex, and imbalanced. The enhanced scalability through parallelization and GPU acceleration allows for efficient handling of high-dimensional ASD datasets, improving prediction speed and model performance. Adaptive boosting mechanisms and advanced regularization techniques help in reducing overfitting, ensuring that the model generalizes well across diverse patient data. The incorporation of SHAP values for model interpretability further aids clinicians in understanding the key features driving the predictions, making XGBoost 2.0 a powerful tool in ASD prediction.

### 4.4 Shapley additive explanations

**Figure 6:**
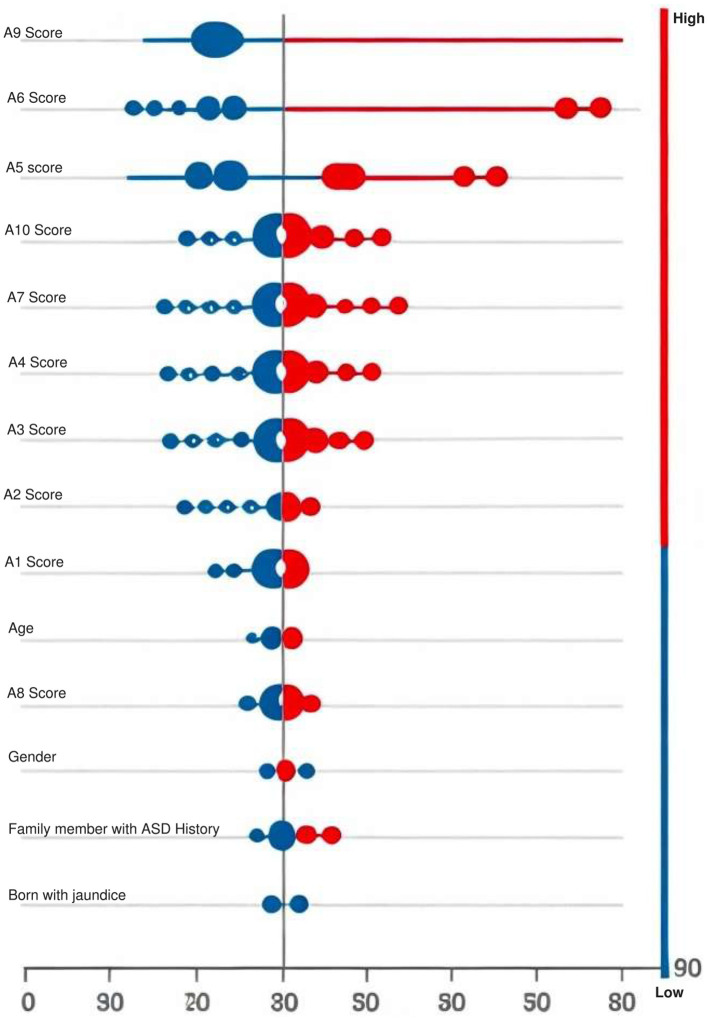
Visual depiction of SHAP feature importance, with color intensity reflecting the significance of each feature; red denotes higher importance, while blue indicates lesser importance.

Understanding how inputs and outputs are related in machine learning (ML) models can be complicated due to their opaque nature. The SHAP technique provides a quantitative approach to measuring model interpretability by evaluating feature importance. It assigns importance values to each feature by assessing its impact on the model's predictions. This technique utilizes cooperative game theory to elucidate complex models. SHAP analysis offers insights into the importance of features for predicting autism spectrum disorder. Beeswarm plots visually display how each feature contributes to individual predictions made by a machine learning model. These plots help us understand which features influence predictions and whether their impact is positive or negative. The SHAP summary (shown in Figure 6) demonstrates the contribution of each significant feature to each individual case within the dataset. Notably, features like A9 score and A6 score emerge as crucial contributors.

### 4.5 Second scenario

**Figure 7:**
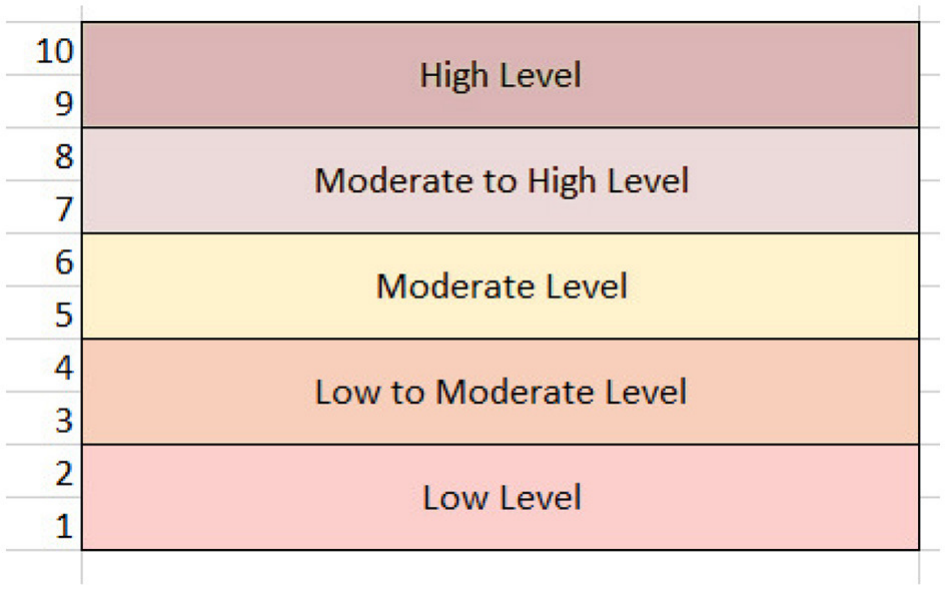
Autism severity levels in children.

This study aims to apply an ML system that can recognize trends in gender, other autism-related characteristics, and autistic symptoms. Estimating the best teaching strategy for each student is the main goal. In this set of experiments, “Teaching Method” named column was created and appended to a dataset with the pandas library. This category comprises integer values ranging from 1 to 6, with the inclusion of the integer zero representing a non-test form, indicating no involvement in special education. This resaerch tailor teaching techniques based on the degree of autistic behavior, which is assessed through the count-based evaluation of severity levels in the Q-Chat-10 score of the dataset, derived from A1-A10 values. Children with higher scores are considered more severely affected, and the severity levels are illustrated in Figure 7.

Table 10 provides the details of the severity level of ASD in children as low level, low to high, low to moderate, moderate, and low level and provides appropriate teaching recommendations for each of these levels.

**Table 10:** Examples of different severity levels.

**Severity level**	**Recommendation**
High-level autistic children	Important Response Training (PRT): Because it places a strong focus on fostering critical skills including initiative, motivation, and social communication, PRT may be beneficial for high-functioning autistic children.
Children with low to high levels of ASD	Leveraging technology-aided instruction can offer significant benefits to low to high-level autism in children for an advanced customized education experience. Customized applications can address individual skill sets, assisting in their enhancement.
Children with moderate-level ASD	The PECS provides invaluable advantages for children with moderate autism by aiding in the overcoming of language barriers through the utilization of visual communication techniques.
Children with low to moderate levels of ASD	Task Analysis: Since task analysis allows the simplifying of skills into definable units, it is appropriate for children who lack important knowledge and require explicitness.
Low-level autistic children	Intensive Behavioral Intervention (IBI): Applied behavior analysis's specialized form of IBI is tested on kids with severe developmental delays, particularly those with low-grade autism.

The model assessment to choose the best teaching approach for children with ASD is made according to the severity levels designed in Table 10. To evaluate the algorithms' correctness, the values of the testing set and the predicted values of the instructional approaches are compared. The results for the ML models are shared in Table 11. The findings indicate that these classifiers undergo predictive performance evaluation to determine optimal teaching method recommendations for autistic children. With an outstanding accuracy score of 0.9929, XGBoost 2.0 stands out as the best-performing model among the classifiers, indicative of its high precision in prediction. XGBoost 2.0 also demonstrates exceptional levels of precision, recall, and F1 scores, all standing at 0.99. Such performance suggests the tool's capability to provide accurate recommendations while effectively capturing a substantial proportion of known relevant instances.

**Table 11:** Classifiers' experimental outcomes for choosing the best strategy of teaching for kids with ASD.

**Models**	**Accuracy**	**Precision**	**Recall**	**F1 score**
SVM	0.9921	0.99	0.99	0.99
RF	0.9912	0.97	0.97	0.97
DT	0.9341	0.93	0.93	0.93
GBM	0.9530	0.95	0.95	0.95
LR	0.9991	0.99	0.99	0.99
XG	0.9821	0.98	0.98	0.98
KNN	0.9534	0.95	0.95	0.95
XGBoost 2.0	0.9929	0.99	0.99	0.99

These factors underscore the value of classifiers in providing relevant and useful recommendations when choosing an effective teaching strategy for kids with ASD. However, using the given evaluation metrics, XGBoost 2.0 consistently shows up as the best model. This knowledge is crucial for educators and professionals working with children with ASD. It enables informed decisions about teaching methods, significantly impacting learning and developmental outcomes.

## 5 Conclusion

Autism spectrum disorder (ASD) presents a multifaceted challenge, impacting a range of cognitive functions such as object classification, language understanding, and communication. While ASD has genetic underpinnings, early diagnosis and treatment can mitigate the necessity for extensive medical interventions and prolonged treatment processes. However, beyond diagnosis, the endeavor to devise effective teaching methodologies for individuals with ASD emerges as a pivotal challenge. Given the vast diversity within the ASD spectrum, each child exhibits a unique constellation of traits and requirements. Recognizing the unique nature of each autistic child emphasizes the importance of personalized approaches tailored to their individual needs and experiences. This research combined two ASD screening datasets focusing on toddlers and utilized chi-square as a feature selection method. After rigorous feature selection methods, a two phase system was developed. In the first phase, several models are trained including proposed XGBoost 2.0, which demonstrated commendable accuracy in identifying ASD. Subsequently, the focus shifted to devising personalized new ways of teaching ASD children, informed by assessments of their verbal, behavioral, and physical activities. The primary objective was to contribute to the development of new teaching methods for children with ASD, leveraging ML to enhance accuracy. The comprehension of ASD expands and machine learning progresses, authors can devise some ways to accurately identify ASD patterns. Acknowledging the characteristics of each ASD child, the quest for optimal teaching methods remains a fluid and ongoing endeavor within autism research and education. While the model achieved high predictive accuracy, there are certain limitations. The chi-square method might not fully capture non-linear relationships between features, and future work could explore more advanced feature selection techniques. Additionally, the dataset used may not represent global diversity, so future research should aim to include larger, more varied datasets to ensure broader applicability. Practical challenges include the high computational cost of SHAP-based explainability, which could be addressed by more efficient methods for real-time applications. Future work should also focus on deploying the model in clinical settings and integrating it with mobile health technologies for better accessibility and expanding its applicability to larger and decentralized datasets through advanced algorithms like federated learning.

## Data Availability

The original contributions presented in the study are included in the article/supplementary material, further inquiries can be directed to the corresponding authors.
